# Transcriptionally Active Human Papillomavirus in Male Genital Lichen Sclerosus, Penile Intraepithelial Neoplasia, and Penile Squamous Cell Carcinoma

**DOI:** 10.1016/j.xjidi.2024.100320

**Published:** 2024-10-26

**Authors:** Georgios Kravvas, Boyu Xie, Aiman Haider, Michael Millar, Hussain M Alnajjar, Alex Freeman, Asif Muneer, Christopher B Bunker, Aamir Ahmed

**Affiliations:** 1 Department of Dermatology, University College London Hospitals NHS Foundation Trust, London, United Kingdom; 2 Centre for Stem Cell and Regenerative Medicince, King's College London, London, United Kingdom; 3 Department of Histopathology, University College London Hospitals NHS Foundation Trust, London, United Kingdom; 4 The Queen's Medical Research Institute, College of Medicine & Veterinary Medicine, University of Edinburg, Edinburgh, United Kingdom; 5 Department of Urology, University College London Hospitals NHS Foundation Trust, London, United Kingdom; 6 Department of Cell & Developmental Biology, University College London, London, United Kingdom

**Keywords:** Carcinogenesis, Human papillomavirus, Microscopy, RNA biology, Squamous cell carcinoma

## Abstract

Penile intraepithelial neoplasia (PeIN) and penile squamous cell carcinoma (PeSCC) are both thought to be associated with male genital lichen sclerosus and human papillomavirus (HPV) infection through dichotomous pathways: (i) undifferentiated PeIN and warty/basaloid PeSCC are thought to be HPV related, whereas (ii) differentiated PeIN and usual PeSCC are considered HPV independent. Tissue arrays were constructed from male genital lichen sclerosus, undifferentiated and differentiated PeIN, usual-type PeSCC, and unaffected tissues. Staining for p16 and for high-risk and low-risk HPV subtypes through RNAscope was performed. The expression of HPV RNA and p16 were quantified, and appropriate statistical comparisons were undertaken. High-risk HPV was prevalent in undifferentiated PeIN (77%) and less so in PeSCC (46%) and was exiguous or absent in all other tissues. LR HPV was only observed in 2 tissue cores. Strong p16 staining exhibited 96.15% sensitivity and 100% specificity for high-risk HPV. Transcriptionally active HPV is unlikely to be implicated in male genital lichen sclerosus and differentiated PeIN, although it is clearly important in undifferentiated PeIN. The high prevalence of high-risk HPV in usual PeSCC challenges the existing paradigm. Strong p16 positivity was a reliable surrogate marker for the detection of transcriptionally active high-risk HPV.

## Introduction

Male genital lichen sclerosus (MGLSc) is a chronic, progressive inflammatory and fibrosing skin disorder that can lead to significant sexual and urological morbidity and predisposes to penile intraepithelial neoplasia (PeIN) and penile squamous cell carcinoma (PeSCC) (Bunker, 2019; Bunker and Porter, 2016; Kravvas et al, 2022a). The evidence points to chronic occlusive exposure to urine in the balanopreputial sac as a key driver for MGLSc pathogenesis (Kravvas et al., 2022b, 2018; Panou et al, 2022). Human papillomavirus (HPV) has also been implicated in the pathogenesis of MGLSc, although its role remains controversial, and the exact relationship to MGLSc still remains unknown (Bunker, 2019; Bunker and Porter, 2016; Kravvas et al., 2022b). PeIN and PeSCC can arise from both MGLSc and HPV infection, but dichotomous pathways have been proposed: (i) undifferentiated PeIN (uPeIN) and warty/basaloid squamous cell carcinoma are thought to be HPV related, whereas (ii) differentiated PeIN (dPeIN) and usual PeSCC are considered HPV independent and related to the presence of MGLSc (Kravvas et al., 2022b; Renaud-Vilmer et al, 2010). However, the exact relationship between MGLSc, PeIN, PeSCC, and HPV remains unclear (Bunker, 2019; Bunker and Porter, 2016; Kravvas et al., 2022a, Kravvas et al., 2022b).

Histologically, uPeIN is defined by the presence of atypia affecting at least two thirds of the epidermis, with disorganized epithelial architecture; dyskeratosis; parakeratosis; enlarged, hyperchromatic, and pleomorphic nuclei; atypical mitoses; and occasional koilocytes. In contrast, dPeIN is characterized by dyskeratosis, acanthosis, and elongated rete ridges. Although the superficial maturation of the epithelium is conserved, there are atypical basal and parabasal keratinocytes containing abundant cytoplasm and hyperchromatic irregular nuclei with some mitotic figures, with occasional squamous pearls at the tip of the rete ridges (Bleeker et al, 2009; Cubilla et al, 2004; Dauendorffer et al, 2017a, 2017b; Mannweiler et al, 2013; Velazquez et al, 2012).

HPV belongs to a family of double-stranded DNA viruses that can infect stratified squamous epithelia. Over 200 types of HPV have been identified and are grouped in 5 genera (α, β, γ, μ, and ν) according to the viral genome structure (de Sanjosé et al, 2018).

From a clinical perspective, HPVs are classified into high-risk and low-risk types. Low-risk HPV (LR HPV) infection can cause warty lesions at multiple cutaneous sites, whereas high-risk HPV (HR HPV) types are known to play a crucial carcinogenic role in cervical, anal, oropharyngeal, and penile carcinoma and in related intraepithelial neoplastic conditions (Bunker and Porter, 2016; Lizano et al, 2009).

There has been a lot of speculation regarding the role of HPV infection in the development of MGLSc (Bunker and Porter, 2016; Bunker and Shim, 2015; Shim et al, 2020). A number of studies have attempted to address the relationship between HPV and MGLSc using mainly antibody-based assays (Bunker and Porter, 2016; Bunker and Shim, 2015; Shim et al, 2020). These attempts have yielded variable results and have not been able to resolve the matter largely owing to the lack of specificity of the assays used.

Recent developments in RNA detection technology in situ have opened up avenues to investigate RNA expression of viruses such as HPV. One such technique is RNAscope. This is an mRNA in situ hybridization technique used for the detection of specific mRNA transcripts within morphologically intact tissue samples (Wang et al, 2012). RNAscope probes hybridize to target mRNA molecules with high specificity and sensitivity and allow for single molecules of mRNA to be labeled in thin tissue sections without destroying the tissue architecture (Wang et al, 2012).

To assess the presence of transcriptionally active HPV in MGLSc, PeIN, and PeSCC, we have used 2 RNAscope probes that detect multiple high- and low-risk types of HPV on archival human tissue samples. This work reports on the presence of transcriptionally active HPV infection in MGLSc, uPeIN, dPeIN, and PeSCC using highly specific probes. We demonstrate that (i) HR HPV is highly expressed in uPeIN, (ii) HR HPV is also expressed in almost half of usual PeSCC, (iii) HR HPV expression is however virtually absent in MGLSc and dPeIN, and (iv) LR HPV was largely absent in any of the samples tested. Our results confound previous observations and raise important questions about the role of HPV in MGLSc, PeIN, and PeSCC.

## Results

### Determination of transcriptionally active HPV

**Figure 1:**
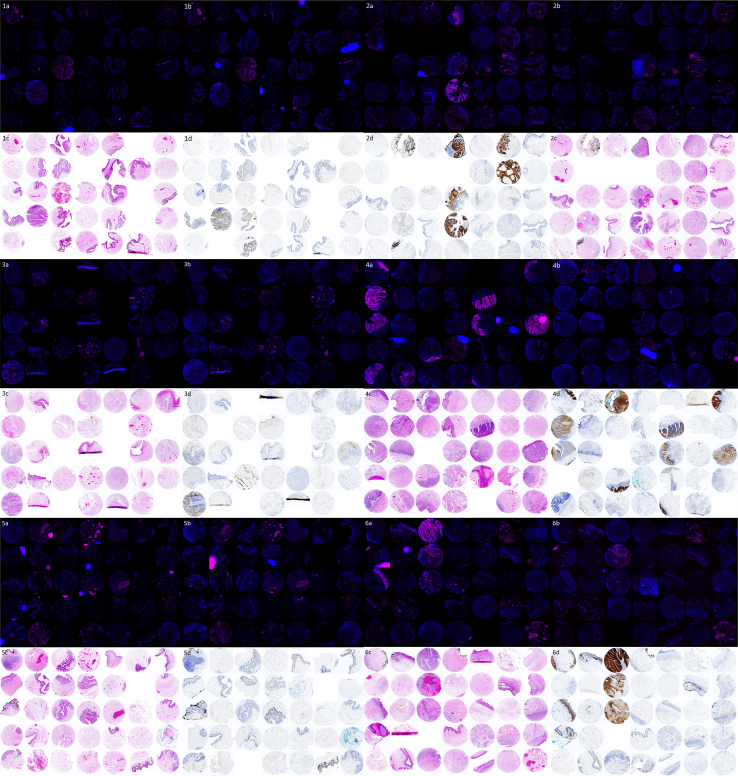
**A composite figure of all the cores examined in this study.** Numerals indicate the number of the pictured tissue array (n = 1–6), and letters indicate the name of the staining method utilized on each sister section. (**a**) HR-HPV18, (**b**) HPVLR10, (**c**) H&E, and (**d**) p16. HPV, human papillomavirus; HR, high risk; LR, low risk.

A total of 129 usable tissue cores were utilized for analysis of LR HPV, and 125 were utilized for analysis of HR HPV. Images of all the tissue cores used in this investigation are given in Figure 1.

**Figure 2:**
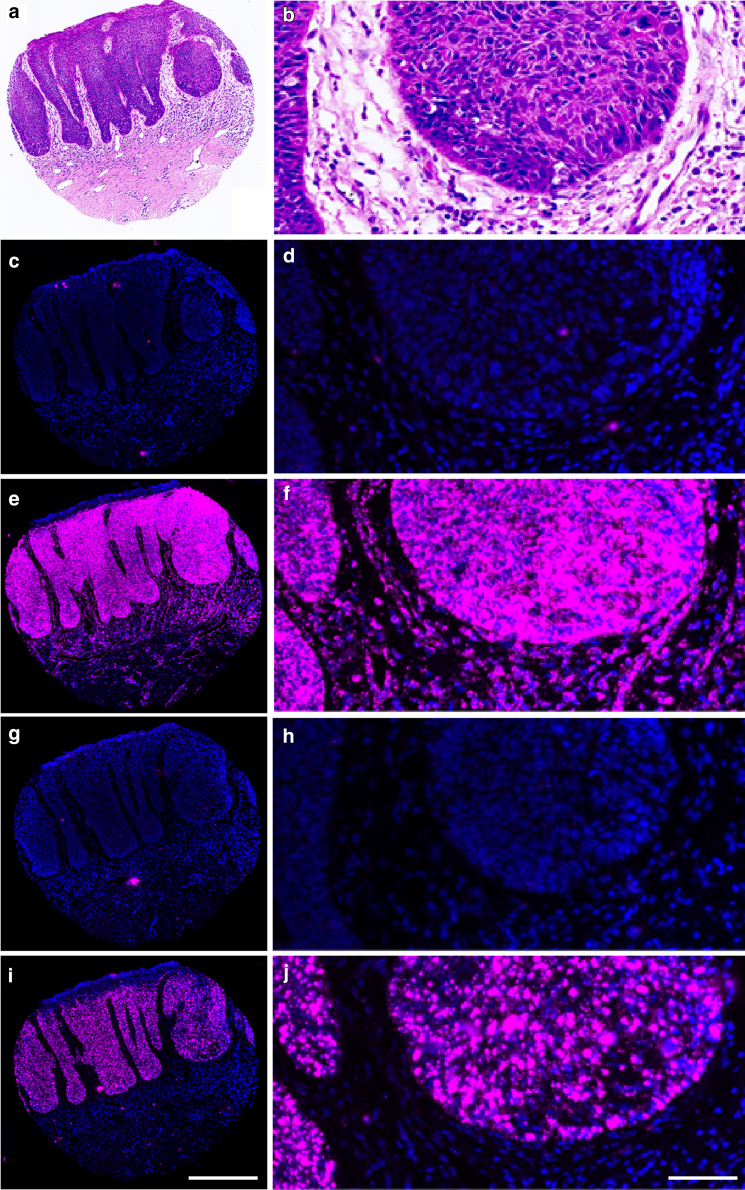
**RNAscope analysis of a HR HPV–positive tissue core.** An example of the analysis of a tissue core stained with H&E and with DAPI (blue, nuclear marker) and Opal 650 (pink) for dapB (negative control), UBC (RNA integrity marker), and 2 moieties of HPV (HR HPV and LR HPV, HPV-HR18, and HPVLR10, respectively). (**a, b**) H&E is used to identify areas corresponding to the epidermis. (**c, d**) The dapB probe displays minimal Opal 650 signal, which is attributable to noise. (**e, f**) The UBC probe displays ample Opal 650 signal, denoting the presence of intact RNA. (**g, h**) The HPVLR10 probe exhibits minimal signal that is comparable with that of the dapB probe, indicating the absence of transcriptionally active LR HPV. (**i, j**) The HPV-HR18 probe exhibits abundant Opal 650 signal in the epidermis and indicates the presence of transcriptionally active HR HPV. HPV, human papillomavirus; HR HPV, high risk human papillomavirus; LR HPV, low risk human papillomavirus. Scale bar = 600 μm for **a,c,e,g,i**; 100 μm for **b,d,f,h,j**.

**Figure 3:**
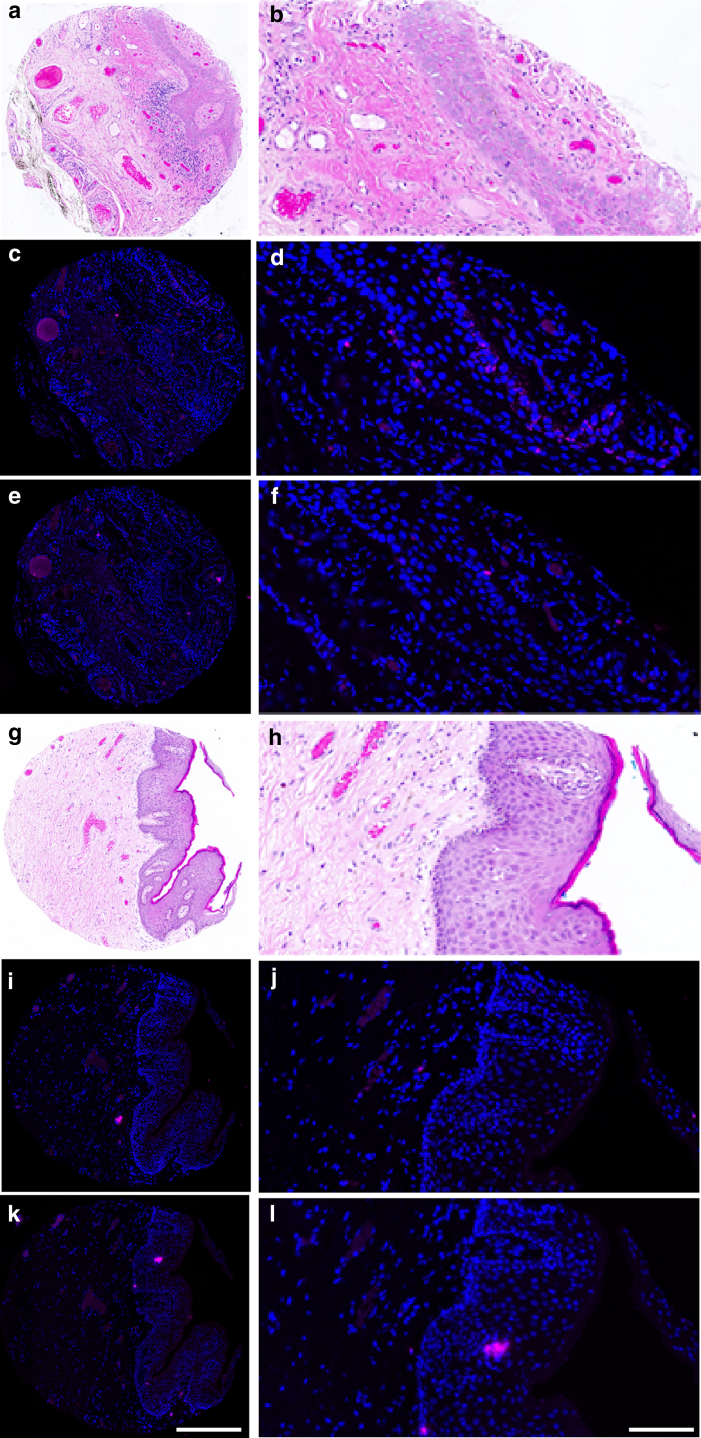
**RNAscope analysis of HR HPV–negative tissue cores.** Examples of the analysis of 2 tissue cores stained with H&E and with DAPI (blue, nuclear marker) and Opal 650 (pink) for 2 moieties of HPV (HR HPV and LR HPV, HPV-HR18, and HPVLR10, respectively). (**a, b, g, h**) H&E is used to identify areas corresponding to the epidermis. (**c, d**) The HPVLR10 probe exhibits Opal 650 signal in the epidermis and indicates the presence of transcriptionally active LR HPV. (**i, j)** The HPVLR10 probe exhibits minimal signal, indicating the absence of transcriptionally active LR HPV. (**e, f, k, l**) The HPV-HR18 probe exhibits minimal signal, indicating the absence of transcriptionally active HR HPV. The analysis of the dapB and UBC probes was performed in line with that in Figure 2c–f and is not displayed in this image for concision. HPV, human papillomavirus; HR HPV, high risk human papillomavirus; LR HPV, low risk human papillomavirus. Scale bar = 600 μm for **a,c,e,g,i,k**; 200 μm for **b,d,f,h,j,l**.

Figures 2 and 3 show examples of the utility of sister sections of the sample stained for H&E, dapB, UBC, HPV-HR18, and HPV-LR10 probes. This approach allowed us to identify the morphology and specificity of the Opal 650 signal and integrity of RNA in the tissue core and to assess for the presence of transcriptionally active HPV. Using this approach, all samples showed good RNA integrity; little to no background, nonspecific signal; and presence of transcriptionally active HPV RNA transcripts.

**Figure 4:**
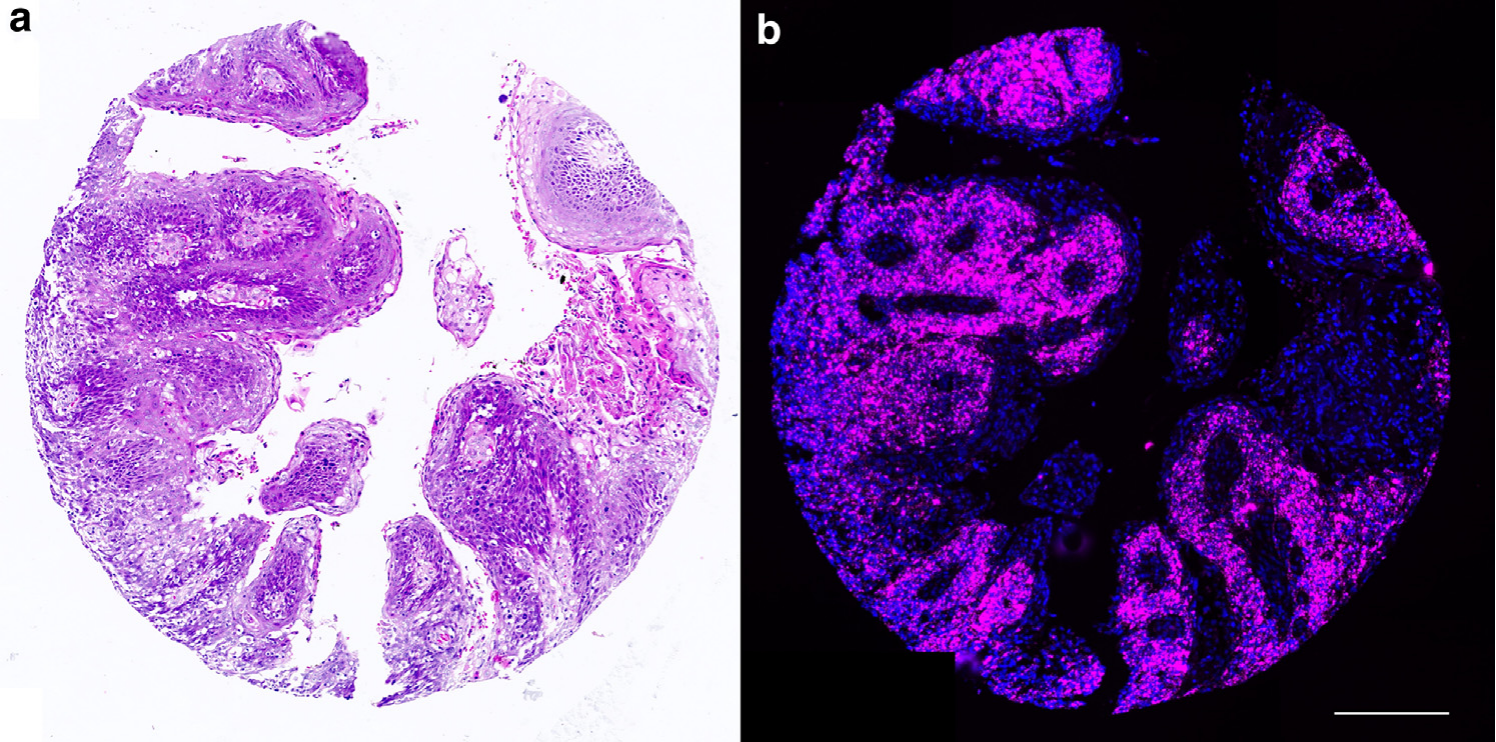
**H&E staining and RNAscope probing of a tissue core.** Micrographs of a single tissue core of PeSCC (**a**) stained with H&E and (**b**) a sister section of exactly the same sample showing the expression of single-molecule RNA probed for HPV-HR18 (pink) and DAPI (nuclear marker, blue). HPV, human papillomavirus; PeSCC, penile squamous cell carcinoma. Scale bar = 600 μm.

A representative example of a H&E-stained tissue core and a corresponding sister section of the same tissue sample stained for an RNAscope probe is shown in Figure 4. There appears to be a good correlation between epidermis as visualized by H&E stain (Figure 4a) and the RNAscope signal (Figure 4b, pink) in the sister sections.

**Figure 5:**
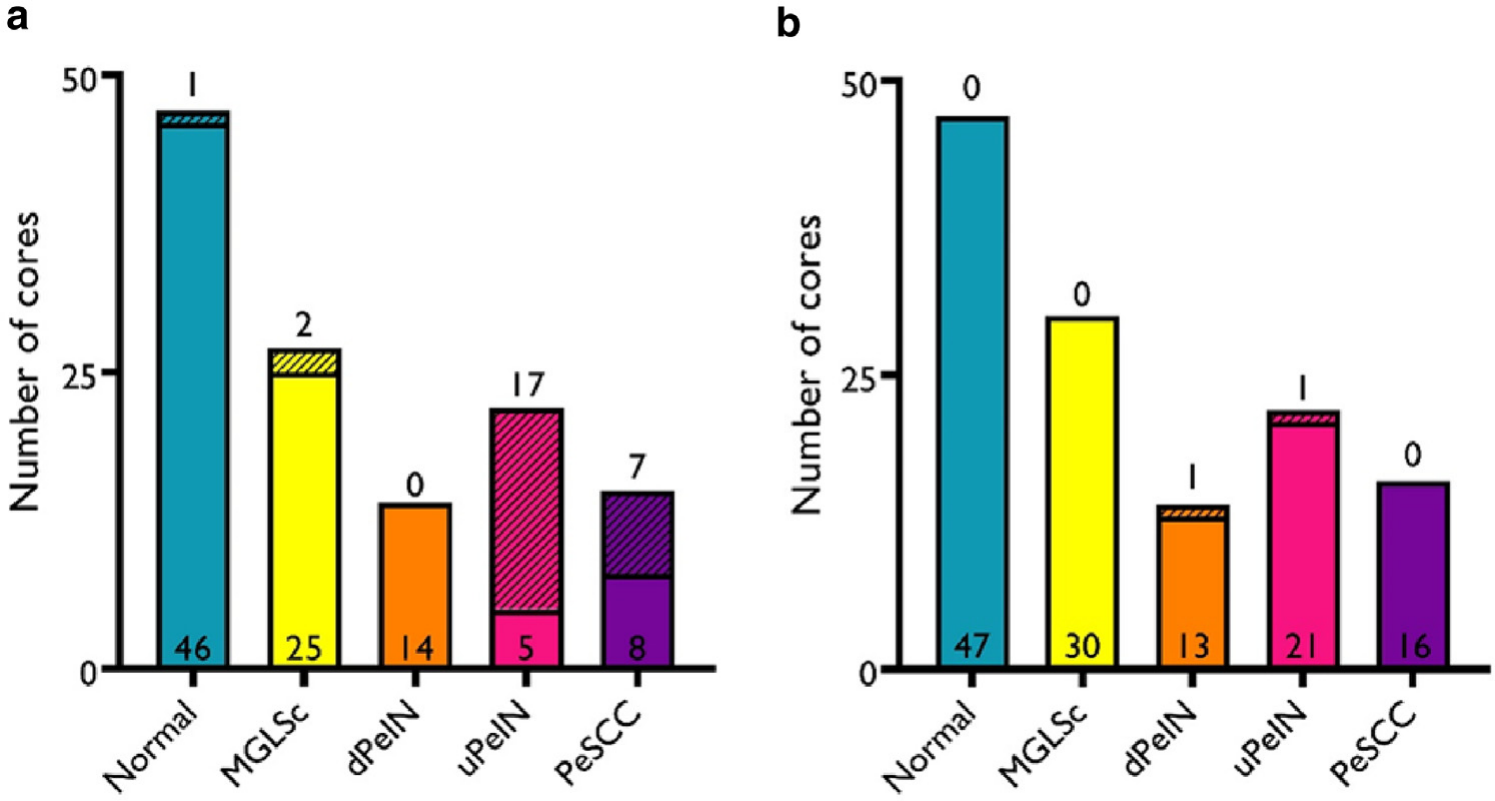
**The number of transcriptionally active HPV cores per condition.** Numbers of positive and negative cores in (**a**) HR HPV and (**b**) LR HPV for the conditions under investigation. Open-colored bars represent HR HPV–negative cores, and hatched-colored bars represent HR HPV–positive cores. Only uPeIN (77.3%) and PeSCC (46.7%) samples have significant numbers of HR HPV–positive samples. The MGLSc set only contains 2 positive samples (7.1%), and the normal set only contains 1 positive sample (4.5%). dPeIN, and uPeIN cores did not contain any positive samples. A total of 125 samples were used for analysis of HR HPV positivity. dPeIN, differentiated penile intraepithelial neoplasia; HPV, human papillomavirus; HR HPV, high-risk human papillomavirus; LR HPV, low-risk human papillomavirus; MGLSc, male genital lichen sclerosus; PeSCC, penile squamous cell carcinoma; uPEIN, undifferentiated penile intraepithelial neoplasia.

The expression for HR HPV single-molecule RNA was thus present in 77% of all uPeIN cores and in 46% of all PeSCC cores. The HR HPV signal was exiguous or absent in the other conditions and in normal tissues (Figure 5a). LR HPV was only observed in 2 tissue cores, 1 of dPeIN (constituting 7% of tissue samples), and 1 of uPeIN (4%); all other tissue sample cores were negative for LR HPV (Figure 5b). These results indicate a clear difference in the correlation between the presence of HR HPV and uPeIN compared with that of PeSCC; they also show that HPV infection from low-risk types is unlikely to be a major contributor or passenger infection for either of these diseases.

### Differences in the prevalence of transcriptionally active HPV

**Figure 6:**
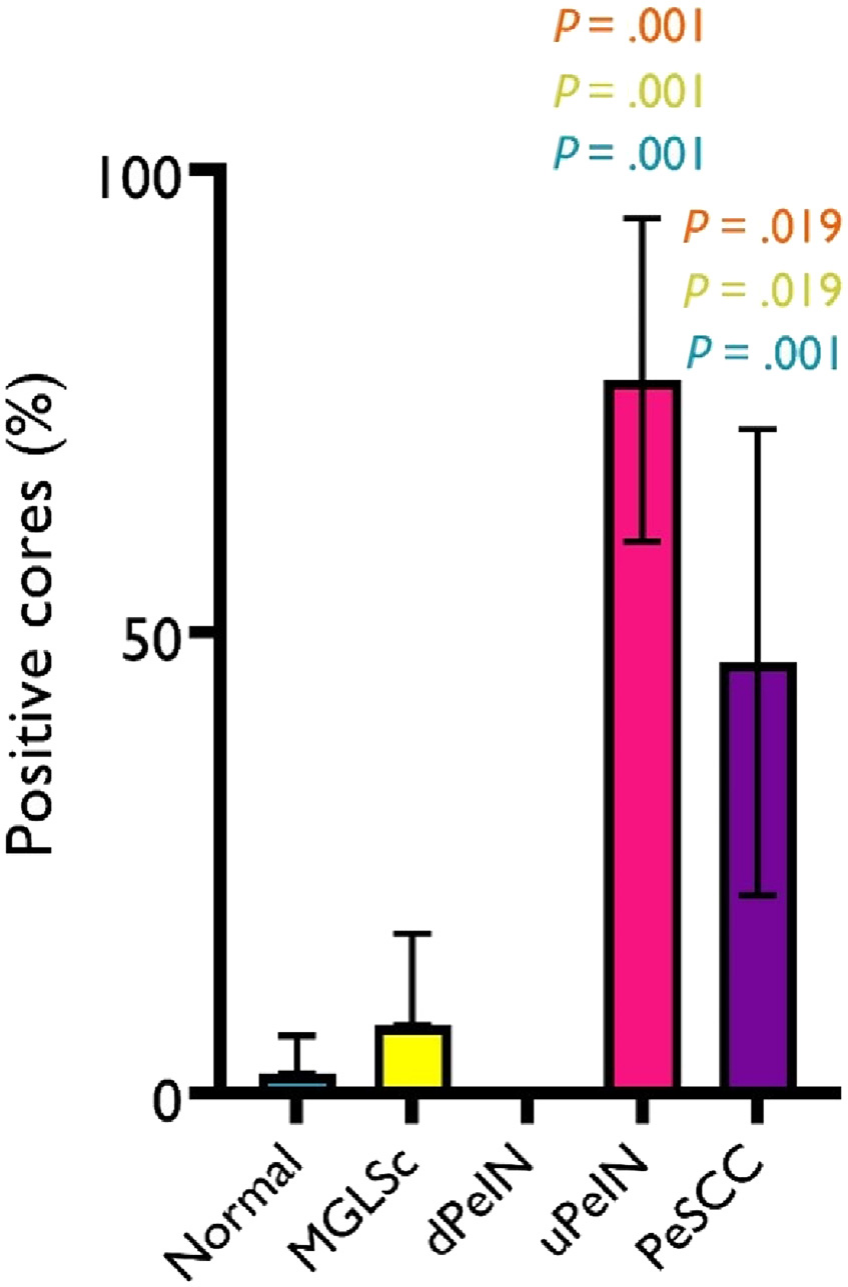
**Comparative statistical analysis of the percentage of HR HPV–positive cores.** Comparison between the normal condition and the various disease states, including MGLSc, dPeIN, uPeIN, and PeSCC, was performed. Statistically significant differences are seen between uPeIN and normal tissues (blue, *P* = .001), MGLSc (yellow, *P* = .001), and dPeIN (orange, *P* = .001). Statistically significant differences are also seen between PeSCC and normal tissues (blue, *P* = .001), MGLSc (yellow, *P* = .019), and dPeIN (orange, *P* = .019). The bars represent the proportions of positive cores with their corresponding 95% confidence intervals. Group comparisons were calculated using the chi-square test with Holm–Bonferroni posthoc test. dPeIN, differentiated penile intraepithelial neoplasia; HR HPV, high-risk human papillomavirus; MGLSc, male genital lichen sclerosus; PeSCC, penile squamous cell carcinoma; uPEIN, undifferentiated penile intraepithelial neoplasia.

Compared with normal tissues, both uPeIN (*P* = .001) and PeSCC (*P* = .001) cores were found to harbor statistically significant differences in HR HPV positivity. The differences between MGLSc and dPeIN with normal tissues were not statistically significant (Figure 6).

Statistically significant differences in HR HPV positivity were seen between MGLSc and both uPeIN and PeSCC (with *P* = .001 and .019, respectively). Equally, statistically significant differences were noted between dPeIN and both uPeIN and PeSCC (with *P* = .001 and .019, respectively). In contrast, there was no statistically significant difference in HR HPV found between MGLSc and dPeIN and between uPeIN and PeSCC (Figure 6).

No further statistical analysis of LR HPV was performed owing to the extremely low percentage of samples that were positive.

### Correlation between p16 and RNAscope

**Figure 7:**
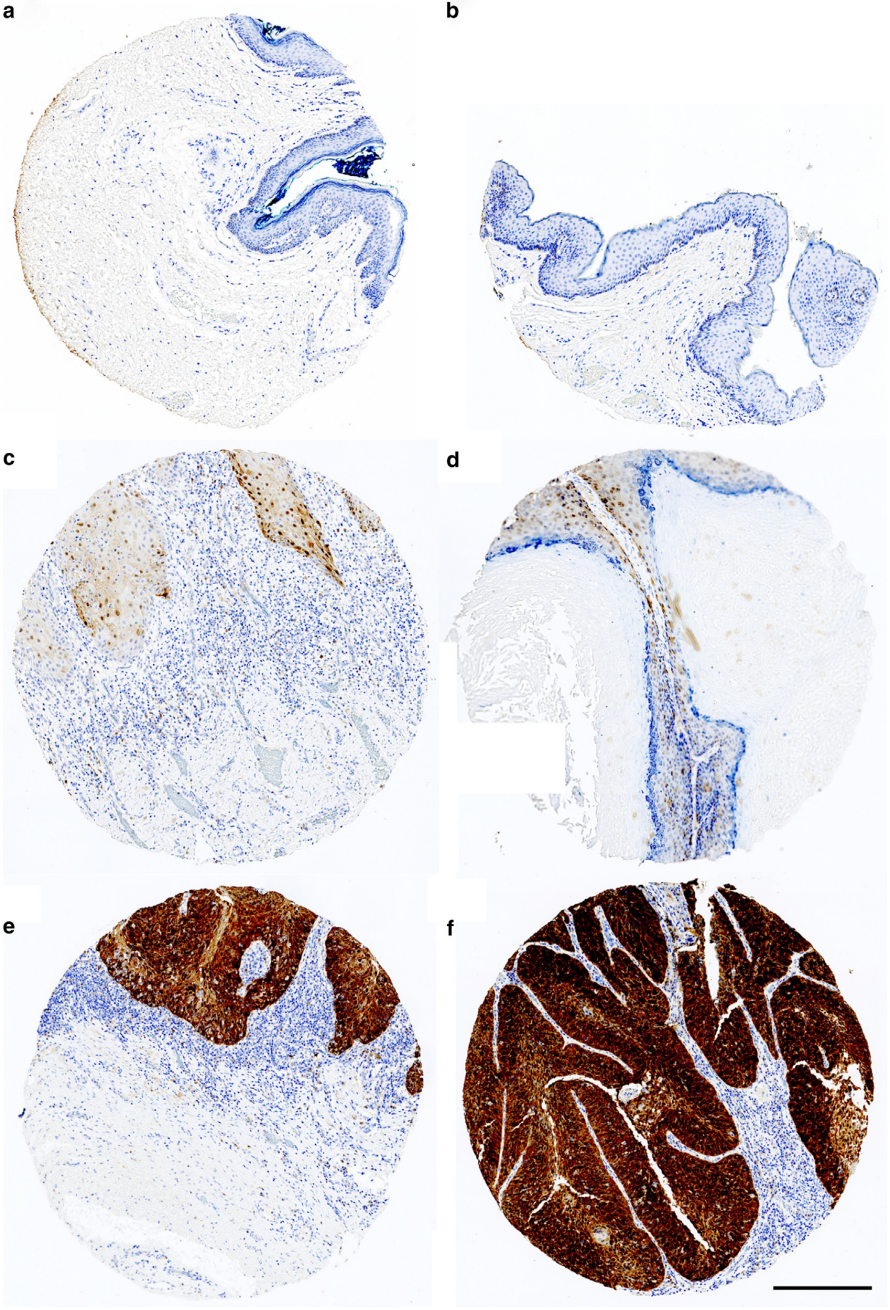
**Examples of p16 staining patterns.** All 6 cores in this image have been stained for p16 (brown). Cores **a** and **b** are negative for p16. Cores **c** and **d** show patchy p16 positivity. Cores **e** and **f** show p16 positivity in a strong, block pattern. Only cores displaying a strong pattern of p16 staining were deemed to be p16 positive. Scale bar = 600 μm.

Of the 124 cores that were usable for assessment of both HR HPV RNAscope and p16 expression, 26 were positive for HR HPV through RNAscope, and 25 showed strong positivity for p16; the remaining cores were negative for both HR HPV and p16. All 25 p16-positive cores were also found to be positive for HR HPV through RNAscope. Only 1 core was found to be positive for HR HPV through RNAscope but negative for p16. In addition, 1 core showed patchy p16 positivity and 1 focal p16 positivity, but neither of these 2 cores were found to be HR HPV positive through RNAscope (Figure 7).

**Figure 8:**
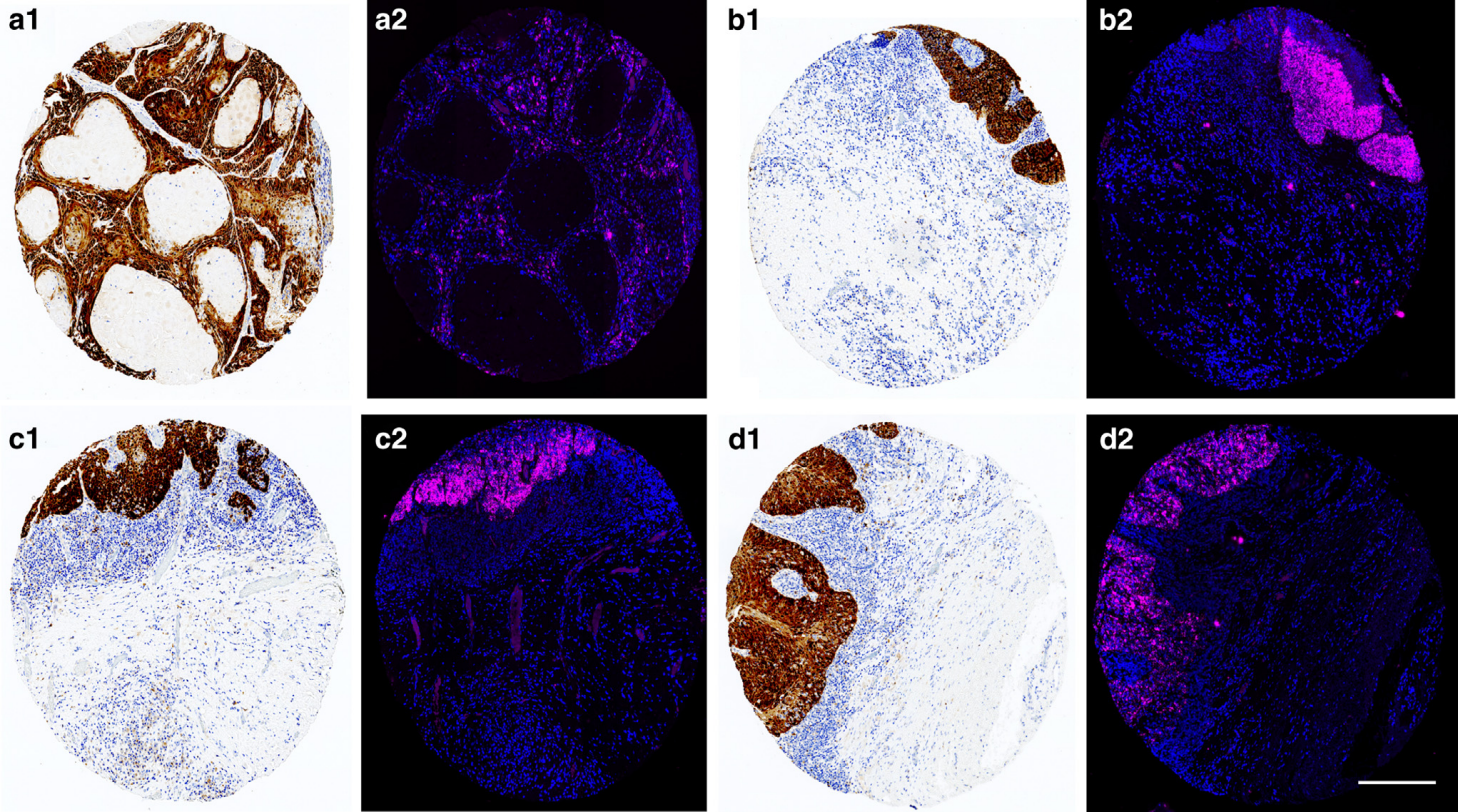
**Correlation between p16 and RNAscope for HR HPV. An example of 4 tissue cores displaying both strong p16 staining and HR HPV RNAscope positivity.** The cores are stained with p16 (brown) (images **a1, b1, c1,** and **d1**) and with DAPI (blue, nuclear marker) and Opal 650 (pink) for HR HPV (HPV-HR18) (images **a2, b2, c2,** and **d2**). A high degree of correlation can be observed in the spatial distribution between p16 staining and HR HPV RNAscope positivity. HR HPV, high-risk human papillomavirus. Scale bar = 600 μm.

Overall, 96% of all RNAscope-positive cores for HR HPV displayed strong p16 positivity (1%, 95% confidence interval [CI] = 9.2–10.8, *P* = .80). Screening for strong p16 staining achieved 96.2% sensitivity (95% CI = 80.4–99.9%) and 100% specificity (95% CI = 96.3–100%). Furthermore, a high degree of spatial correlation was observed between p16 expression and HR HPV positivity through RNAscope. Examples of the high degree of spatial correlation in HR HPV detection through RNAscope and p16 are given in Figure 8.

A total of 127 cores were usable for assessment of both LR HPV RNAscope and p16. Of them, 25 cores were p16 positive, and only 1 core was also positive for LR HPV RNAscope. Of the 2 LR HPV RNAscope-positive cores, only 1 core was also positive for p16. The difference in positivity between p16 and RNAscope for LR HPV was found to be statistically significant (18.9%, 95% CI = 11.8–27.3%, *P* < .001).

Overall, positivity for p16 was strongly correlated with the presence of transcriptionally active HR HPV on RNAscope but poorly correlated with the presence of LR HPV.

## Discussion

This report allows for direct correlations between the diseases under investigation and infection with transcriptionally active HPV. We show that (i) HR HPV strongly correlated with uPeIN and was present in almost half of all PeSCC cores, (ii) HR HPV showed little correlation with MGLSc and dPeIN, (iii) LR HPV did not show significant correlation with any disease, (iv) transcriptionally active HPV infection correlates well with strong p16 expression in penile tissues. We discuss our results in the framework of these key observations.

### LR HPV

In our experiments, LR HPV was found to be rarely expressed in uPeIN (4%) and dPeIN (7%) and was altogether absent in MGLSc, PeSCC, and all normal tissue samples.

LR HPV types are usually related to common warts but have also been associated with both malignant and premalignant lesions, albeit far less frequently so than HR HPV types have (D'Hauwers et al, 2012; Egawa and Doorbar, 2017). HPV6, specifically, is one of the strains that was included in the low-risk panel in this work and represents the commonest cause of anogenital warts (Arima et al, 2010; Aubin et al, 2008; Krzyzek et al, 1980; Padel et al, 1990). Although none of the patients in this study was noted to have viral warts, either clinically or histologically, the presence of small, inconspicuous anogenital warts cannot be fully excluded.

Whether attributable to inconspicuous warts or not, the exiguous detection of LR HPV in this work is likely to be incidental and highly unlikely to contribute significantly to the pathogenesis of MGLSc, PeIN, or PeSCC.

### HPV in MGLSc

The literature contains variable rates of HPV in MGLSc. Shim et al (2020) detected a combination of HR HPV and LR HPV in 37.5% of patients with MGLSc (n = 88), with HPV16 being the most prevalent HPV type detected (12.5%). Guerrero-Setas et al (2013) identified high-risk HPV66 in only 1 patient with MGLS (3.7%, n = 27), whereas Prowse et al (2008) found HR HPV in 33% of MGLSc (n = 18), and von Krogh et al (2002) found HR HPV in 19% (n = 19) of MGLSc. Nasca et al (2006) identified the presence of HR HPV in 17.4% of all MGLSc (n = 46; HPV16 = 6, HPV18 = 1, HPV45 = 1). Finally, Zhang et al (2016) did not find any positive HR HPV or LR HPV in patients with MGLSc (n = 47).

In contrast to all of the studies mentioned earlier, which have sought the presence of HPV using DNA-based techniques, our work utilized RNA in situ hybridization with RNAscope. This is a specific and sensitive method that detects the presence of transcriptionally active RNA from active infections, whereas DNA-based techniques are also likely to detect dormant DNA or represent contamination (Hammer et al, 2022). Furthermore, the RNAscope panel deployed in this work was designed to only detect the 10 commonest LR HPV subtypes and may have missed rarer infection with some of the hundreds of LR HPVs that have been identified (Burley et al, 2020; McBride, 2022).

The higher rates of HPV reported by previous studies are likely to reflect the detection of idle HPV DNA or the presence of rare and low-risk types of HPV with unclear clinical significance. These reports contribute toward the notion that genital HPV infection is widespread, albeit quiescent, and could be attributed to the disrupted epithelium that is often present in MGLSc. This is an important distinction that may help in molecular delineation of HPV infection and its role for causation of MGLSc, PeIN, and PeSCC.

The very low prevalence of HPV in MGLSc in this work and the lack of statistically significant differences between MGLSc and normal penile skin (*P* = .8) suggest that HPV is unlikely to be driver of the pathogenesis of MGLSc.

### HPV in PeIN

In the current dichotomous theory of PeIN, uPeIN is considered to be HR HPV driven (Kravvas et al., 2022a). This view is supported by the well-established presence of koilocytes on histopathology and strong p16 positivity on immunohistochemistry (Kravvas et al., 2022a).

Correspondingly, in this work, HR HPV was found in the vast majority of uPeIN (77%) but was completely absent in dPeIN and almost absent in normal tissues. The high prevalence of oncogenic HPV in uPeIN (indeed the highest seen among all the clinicopathological groups investigated) strongly supports the existing paradigm. In addition, however, 23% of uPeIN did not show expression of HPV and therefore might have different mechanisms of carcinogenesis. This is a key discovery from this research and can have both fundamental and clinical relevance to the understanding of the disease process and the mechanisms of penile carcinogenesis.

Furthermore, the current opinion holds that dPeIN is largely driven by MGLSc and not associated with oncogenic HPV (Bunker and Porter, 2016; Kravvas et al., 2022b). Indeed, the complete absence of any HR HPV in dPeIN in this work adds weight to the existing theory.

### HPV in PeSCC

The carcinogenic process that leads to usual PeSCC is thought to be HPV independent, and instead believed to arise from MGLSc via dPeIN (Moch et al, 2016; Ro et al, 2020).

In this work, HR HPV was seen in almost half of all cases of usual PeSCC (46.7%). Furthermore, important differences were seen when comparing the prevalence of HR HPV between PeSCC and its non-HPV–driven presumed precursors, namely MGLSc (*P* = .019) and dPeIN (*P* = .019).

The high prevalence of HR HPV seen in usual PeSCC challenges the current dichotomous pathway paradigm. It suggests that HR HPV may somehow be implicated in usual PeSCC carcinogenesis and that this pathway is not a purely MGLSc/dPeIN-driven process. This evidence is important both for fundamental etiopathogenesis and for the clinical management of the disease.

### HPV and p16

p16 is thought to be a tumor-suppressor protein, coded by the *CDKN2A* gene, which under normal circumstances regulates the cell cycle and slows cell division. However, in HPV-driven cancers, p16 has been shown to be overexpressed (Sritippho et al, 2016). This overexpression occurs because the HR HPV E7 oncoprotein suppresses the retinoblastoma tumor-suppressor pathway, rendering the cell cycle insensitive to p16 repression (Martin et al, 1998). p16 immunohistochemistry is thus used as a marker for HPV positivity in several precancer and cancers, including cervical carcinoma, PeIN, and PeSCC (Bunker and Porter, 2016; Kravvas et al., 2022a; Sritippho et al, 2016).

The estimated analytical sensitivity of p16 as a surrogate marker for HPV infection ranges from 40 to 100%, and the specificity ranges from 66 to 81% (Laco et al, 2012; Martins et al, 2018; Śnietura et al, 2010; Sritippho et al, 2016; Zito Marino et al, 2021). However, these numbers vary greatly between publications depending on the type of malignancy studied and the methods used to detect the presence of HR HPV (mainly through PCR techniques for identification of the DNA of HR HPV subtypes) (Laco et al, 2012; Martins et al, 2018; Śnietura et al, 2010).

In a study from 2021, Marino et al (2021) compared p16 high-intensity RNAscope in patients with PeSCC of different subtypes. They found that as surrogate of HR HPV positivity, high-intensity p16 staining achieved 89% sensitivity and 100% specificity (Marino et al, 2021). Our study found even higher levels of sensitivity of high-risk RNAscope and strong p16 expression (96% sensitivity and 100% specificity), thus supporting the use of p16 expression as a surrogate marker for transcriptionally active HR HPV. Our study provides an independent validation of p16 as a surrogate marker for HPV infection, and our results indicate a strong correlation between the presence of transcriptionally active HPV and p16 expression in the same tissue samples.

This work has produced evidence about the role of HPV in MGLSc, PeIN, and PeSCC. More specifically, it supports that transcriptionally active HPV is highly unlikely to be implicated in MGLSc and dPeIN although clearly important in uPeIN. Furthermore, the high prevalence of HR HPV in usual PeSCC challenges the clear-cut dichotomous pathway paradigm. Finally, this work adds evidence in support of strong p16 positivity as a reliable surrogate marker for the detection of HR HPV.

## Materials and Methods

### Ethical approval and sample collection

Ethical approval (REC ref 20/SC/0037) for this study was granted by the joint research office at University College London Hospitals and University College London through the NHS Health Research Authority, South Central – Berkshire B Research Ethics Committee.

Archival, formalin-fixed and paraffin-embedded penile tissue samples from adult patients were collected for this study. Five conditions of interest (MGLSc, dPeIN, uPeIN, PeSCC, and normal [disease adjacent, nonpathological areas]) were identified on tissue sections with the help of 2 expert histopathologists.

The tissues were originally collected from patients who had undergone either circumcision or excision of pathological penile lesions at University College London Hospitals as part of their routine management. All patients gave written informed consent to access of their medical records and tissue samples. Clinical and demographic information was collected and is presented in Table 1.

**Table 1:** Detailed Patient Demographics

Patient Identifier	Diagnosis	PeSCC Subtype	Age at Diagnosis, y
A001	MGLSc	n/a	40
A002	MGLSc	n/a	63
A003	MGLSc	n/a	59
A004	MGLSc	n/a	37
A005	MGLSc	n/a	48
A006	MGLSc	n/a	56
A007	MGLSc	n/a	71
A010	MGLSc	n/a	61
A011	MGLSc	n/a	88
A012	MGLSc	n/a	29
A014	MGLSc	n/a	25
A015	MGLSc	n/a	34
A017	MGLSc	n/a	63
A018	MGLSc	n/a	61
A020	MGLSc	n/a	41
A021	MGLSc	n/a	54
A022	MGLSc	n/a	40
A023	MGLSc	n/a	56
A024	MGLSc	n/a	51
A026	MGLSc	n/a	53
A030	MGLSc	n/a	31
A031	MGLSc	n/a	62
A032	MGLSc	n/a	56
A033	MGLSc	n/a	68
A039	MGLSc	n/a	44
A040	MGLSc	n/a	47
A041	MGLSc	n/a	49
A042	MGLSc	n/a	82
A043	MGLSc	n/a	80
A045	MGLSc	n/a	42
A046	MGLSc	n/a	55
A049	MGLSc	n/a	33
A051	MGLSc	n/a	39
A052	MGLSc	n/a	24
A053	MGLSc	n/a	50
A055	MGLSc	n/a	51
A058	MGLSc	n/a	78
A060	MGLSc	n/a	47
A062	MGLSc	n/a	79
A064	MGLSc	n/a	73
A071	MGLSc	n/a	67
A075	MGLSc	n/a	48
A076	MGLSc	n/a	71
A077	MGLSc	n/a	55
A078	MGLSc	n/a	71
A079	MGLSc	n/a	51
A080	MGLSc	n/a	53
A081	MGLSc	n/a	79
A010	uPeIN	n/a	61
B003	uPeIN	n/a	56
B006	uPeIN	n/a	53
B008	uPeIN	n/a	78
B009	uPeIN	n/a	51
B015	uPeIN	n/a	59
B016	uPeIN	n/a	70
B030	uPeIN	n/a	82
B030	uPeIN	n/a	83
B032	uPeIN	n/a	24
B034	uPeIN	n/a	67
D012	uPeIN	n/a	90
B035	uPeIN	n/a	51
B036	uPeIN	n/a	44
B041	uPeIN	n/a	60
B046	uPeIN	n/a	53
B065	uPeIN	n/a	69
B076	uPeIN	n/a	53
B080	uPeIN	n/a	47
D006	uPeIN	n/a	32
D007	uPeIN	n/a	69
D012	uPeIN	n/a	91
B002	dPeIN	n/a	47
B003	dPeIN	n/a	48
B004	dPeIN	n/a	82
B005	dPeIN	n/a	39
B006	dPeIN	n/a	73
B007	dPeIN	n/a	71
B008	dPeIN	n/a	67
B011	dPeIN	n/a	69
B012	dPeIN	n/a	72
B016	dPeIN	n/a	71
B017	dPeIN	n/a	55
B018	dPeIN	n/a	71
B019	dPeIN	n/a	51
B023	dPeIN	n/a	51
B024	dPeIN	n/a	51
B025	dPeIN	n/a	79
B027	dPeIN	n/a	66
B028	dPeIN	n/a	77
B029	dPeIN	n/a	81
B031	dPeIN	n/a	67
B032	dPeIN	n/a	48
D007	PeSCC	Usual	32
D008	PeSCC	Usual	78
D015	PeSCC	Usual	59
D016	PeSCC	Usual	70
D020	PeSCC	Usual	48
D030	PeSCC	Usual	83
D034	PeSCC	Usual	67
D035	PeSCC	Usual	51
D044	PeSCC	Usual	73
D049	PeSCC	Usual	82
D051	PeSCC	Usual	67
D060	PeSCC	Usual	51
D064	PeSCC	Usual	64
D069	PeSCC	Usual	51
D071	PeSCC	Usual	54
D074	PeSCC	Usual	77
D077	PeSCC	Usual	69
D079	PeSCC	Usual	61
D080	PeSCC	Usual	62
D081	PeSCC	Usual	72
D086	PeSCC	Usual	48
D087	PeSCC	Usual	83

Abbreviations: dPeIN, differentiated penile intraepithelial neoplasia; MGLSc, male genital lichen sclerosus; n/a, not applicable; PeSCC, penile squamous cell carcinoma; uPEIN, undifferentiated penile intraepithelial neoplasia.

Presented is a summary table of all study subjects, with associated diagnoses and age at the time of diagnosis given for each patient.

Archival tissue samples from a total of 114 patients were obtained from 48 men with MGLSc, 21 with dPeIN, 22 with uPeIN, and 23 with PeSCC. All PeSCCs included in this study were of the usual subtype. However, for optimal intelligibility, this qualifier has been largely omitted from the rest of this report. The demographics of each group are presented in Table 2.

**Table 2:** Patient Demographics according to Condition

Condition	Median Age of Diagnosis, y	25 and 75% Interquartile Range
MGLSc	53	43–65
dPeIN	69	51–73
uPeIN	60	51–70
PeSCC	66	51–73

Abbreviations: dPeIN, differentiated penile intraepithelial neoplasia; MGLSc, male genital lichen sclerosus; PeSCC, penile squamous cell carcinoma; uPEIN, undifferentiated penile intraepithelial neoplasia.

Tissue samples were obtained from patients with MGLSc (N = 48, n =48), dPeIN (N = 21, n = 25), uPeIN (N = 22, n = 28), and PeSCC (N = 23, n = 23). The mean age of diagnosis and associated interquartile ranges are provided for each condition (N = number of patients; n = number of tissue cores).

### Tissue array construction

A preliminary sample size calculation was conducted for binary outcomes (ie, negative or positive) with a significance level (α = 5%) and power (1-β = 90%) with a percentage success in control group (5%) and percentage success in experimental group (75%). The calculated sample size required was 6 per group (12 total).

A manually operated tissue arrayer (MAT1, Beecher Instruments) was used to construct tissue arrays using previously described techniques (Symes et al, 2013; Xie et al, 2023). Briefly, individual tissue cores (1 mm in diameter) were extracted from the marked regions of MGLSc, dPeIN, uPeIN, PeSCC, and disease-adjacent (normal) penile tissues. Between 1 or 2 individual cores were extracted from each tissue block. These donor cores were then deposited into 6 recipient wax blocks of up to 35 individual tissue cores per block.

Tissue array blocks were sectioned at 5-μm thickness on glass slides using an automatic microtome (HM355S, Thermo Fisher Scientific).

### RNAscope single-molecule RNA detection

RNAscope is a proprietary assay from ACD. The probes selected were able to identify the 16 most common HR HPVs (HPV-HR18, 312598, ACD) and the 10 most common LR HPVs (HPV-LR10, 314558, ACD). *UBC*, a highly transcribed and expressed gene in human tissues, was used as a positive control (Hs-UBC, 312028, ACD), whereas *dapB*, a bacterial gene that is not expressed in human tissues, was used accordingly as the negative control (dapB, 312038, ACD) (Bianchi et al, 2019; Pavelka et al, 1997). The subtypes of HR HPV and LR HPV that could be detected and their associated target genes are shown in Table 3.

**Table 3:** Description of Target Genes and Corresponding RNA Probes

Probe	Name, Catalog Number, Source	Target Gene
HPV-HR18	2.5 LS probe- HPV-HR18, 312598, ACD	*E6*/*E7* genes of HPV16, 18, 26, 31, 33, 35, 39, 45, 51, 52, 53, 56, 58, 59, 66, 68, 73, and 82
HPVLR10	2.5 LS probe- HPVLR10, 314558, ACD	*E6*/*E7* genes of HPV6, 11, 40, 43, 44, 54, 69, 70, 71, and 74
Hs-UBC	2.5 LS positive control probe- Hs-UBC, 312028, ACD	342–1503 region of human *UBC* gene
dapB	2.5 LS negative control probe-dapB, 312038, ACD	414–862 region of nonhuman *dapB* gene

Abbreviations: HPV, human papillomavirus; HR HPV, high-risk human papillomavirus; LR HPV, low-risk human papillomavirus.

The table provides a summary of the RNA probes used in this study and the corresponding gene transcripts. HPV-HR18 is designed to target the *E6*/*E7* genes from 18 HR HPV types, and HPVLR10 is designed to target the *E6*/*E7* genes from 10 LR HPV types. Hs-UBC targets the ubiquitous human *UBC* gene and acts as a positive control for measuring tissue RNA integrity, and dapB targets the nonhuman *dapB* gene and acts as a negative control to assess nonspecific label signal.

These probes were used according to the manufacturer’s instructions to investigate the integrity of RNA, contamination from bacterial sources, and specific RNA moieties in human penile tissues. Appropriate RNAscope probe optimization was performed using the recommended hs-UBC (positive control for measuring RNA integrity) and dapB (negative control for background signal removal) probes.

For in situ mRNA detection, RNAscope LS2.5 brown probes were used as per the manufacturer’s instructions. RNAscope staining was visualized using either 3,3′-diaminodbenzidine to establish protease and HIER conditions or alternatively by substituting a fluorescent tyramide at 1:100 dilution for a fluorescent endpoint used for sample analysis (eg, Opal 650, Akoya Biosystems). DAPI was used as a counterstain.

### Histology and detection of p16 expression

For histology, additional sister sections to those used for RNAscope staining were stained with H&E.

p16 protein detection is used as surrogate for HPV presence (da Mata et al, 2021). To address the correlation of p16 protein expression with transcriptionally active HPV detected using RNAscope, anti-p16 antibody was also utilized (INK4A, 1:100 dilution, 684105, Cell Signaling Technology). Antibody staining on tissue arrays was performed as described elsewhere in detail (Symes et al, 2013; Wang et al, 2012, 2010). This antibody was optimized for pH and concentration dependence, antigen retrieval, and temperature parameters. All staining was carried out on a Leica RX research staining robot.

### Tissue core imaging

The H&E-stained slides were scanned at ×20 magnification with brightfield illumination using an AxioScan Z1 (Zeiss) slide scanner. Each individual core was then re-examined by a histopathologist to ensure the diagnosis. The AxioScan Z1 was also used for the scanning of the slides stained for single-molecule RNA using RNAscope with ×20 magnification. Light intensity and camera exposure times were optimized for each fluorophore and kept constant for all subsequent imaging.

### Image analysis for RNAscope

**Figure 9:**
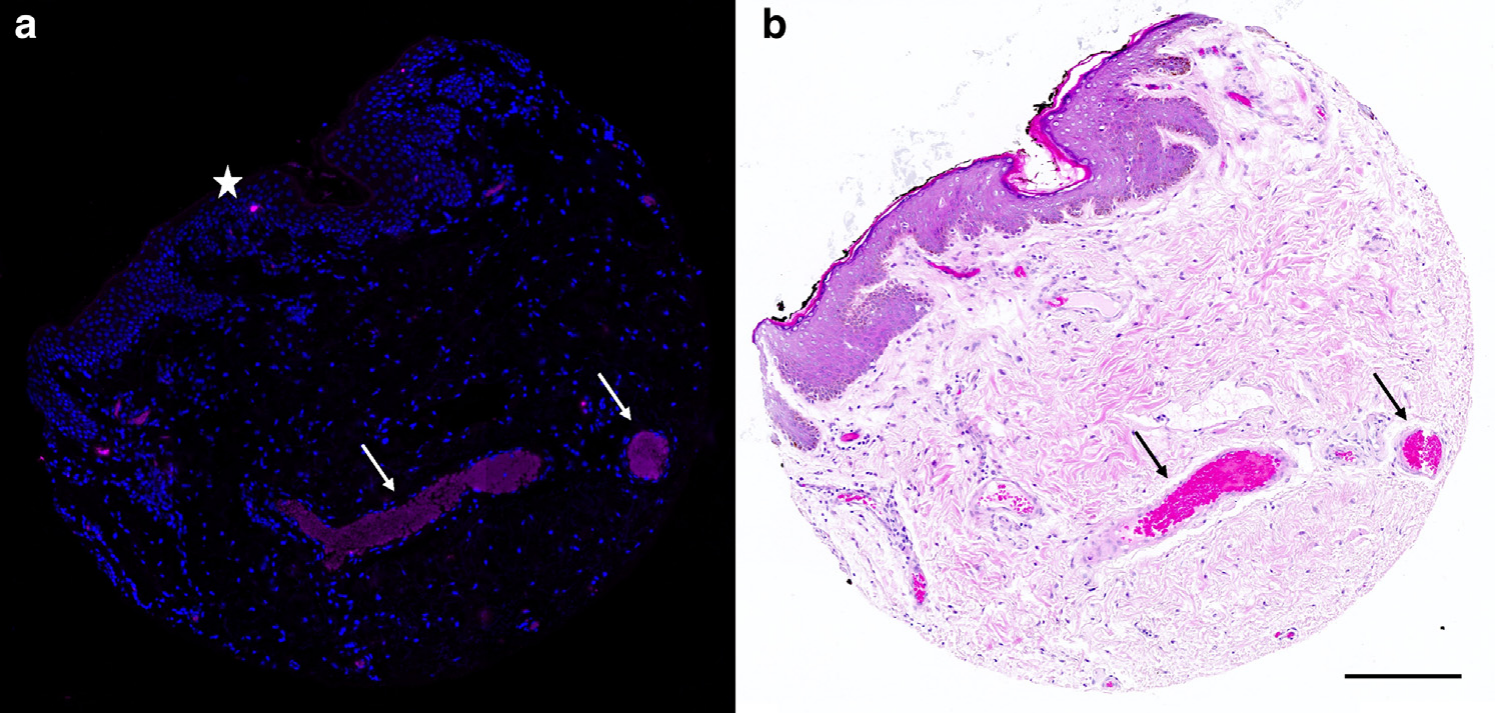
**An example of fluorescing artefact in an HR HPV-negative core**. Sister sections of a single tissue core of normal penile skin stained with (**a**) HPV-HR18 (pink), and DAPI (nuclear marker, blue), and (**b**) H&E. Fluorescing structures due to artefact are shown (**a**) (star and arrows). The structure indicated by the arrows can clearly be identified as blood on H&E (**b**). The patterns of fluorescence seen here are markedly different to that of fluorescing HPV moieties, and were thus considered to be HPV-independent. Scale bar = 600 μm.

Each tissue core was analyzed individually using Zeiss Zen 3.7 software; all RNAscope cores were analyzed visually for the presence of Opal 650 signal representing single-molecule RNA. The UBC signal was viewed first to establish whether mRNA with suitable integrity was present in the tissue samples. Next, using the negative control probe (dapB), thresholding was performed to subtract nonspecific background signal. The thresholding values were applied to the 2 HPV probes that were then assessed for the presence of mRNA signals. The distinctive signal produced from autofluorescing structures, such as blood and gland secretions, or owing to apparent artefact were also eliminated, manually (Figure 9). Cores found bearing the characteristic punctate dot signal of RNAscope were considered to contain transcriptionally active HPV RNA.

### Image analysis for p16

Each tissue core was analyzed independently using Zen 3.7 software. All the cores were assessed manually with a visual analysis for p16 positivity. Thus, only block staining due to strong nuclear and cytoplasmic p16 expression in a continuous segment of cells (at least 10–20 cells) involving the basal and parabasal layers was considered to be positive. Cytoplasmic only staining, diffuse weak intensity staining, and other patchy or focal patterns were considered negative (Darragh et al, 2012).

### Statistical analysis

Binary data are presented in the text and figures as absolute values, proportions, and percentages. Statistical differences were determined using the chi-square test and are presented as *P*-values followed by 95% CIs. The null hypothesis was rejected if the *P* < .05. Owing to the multiple comparisons performed on the data, statistical correction was applied on all *P*-values, using the Holm–Bonferroni method. All statistical tests were undertaken with MedCalc Statistical Software, version 19.2.6 (MedCalc Software, Ostend, Belgium; https://www.medcalc.org; 2020).

## Ethics Statement

Donors of human materials provided written, informed consent. Ethical approval (REC ref 20/SC/0037) for this study was granted by the joint research office at University College London Hospitals and University College London through the NHS Health Research Authority, South Central – Berkshire B Research Ethics Committee.

## Data Availability Statement

All data needed to replicate the study are available within the article.

## ORCIDS

Georgios Kravvas: http://orcid.org/0000-0002-1924-0149

Boyu Xie: http://orcid.org/0000-0002-3966-3261

Aiman Haider: http://orcid.org/0000-0002-7005-4245

Michael Millar: http://orcid.org/0000-0003-4264-3568

Hussain M. Alnajjar: http://orcid.org/0000-0001-6364-0310

Alex Freeman: http://orcid.org/0000-0001-5031-3791

Asif Muneer: http://orcid.org/0000-0003-2958-1614

Christopher B. Bunker: http://orcid.org/0000-0002-6693-7483

Aamir Ahmed: http://orcid.org/0000-0001-7405-5336

## Conflict of Interest

The authors state no conflict of interest
